# Fabrication and Modelling of a Reservoir-Based Drug Delivery System for Customizable Release

**DOI:** 10.3390/pharmaceutics14040777

**Published:** 2022-04-02

**Authors:** Margarethe Hauck, Jan Dittmann, Berit Zeller-Plumhoff, Roshani Madurawala, Dana Hellmold, Carolin Kubelt, Michael Synowitz, Janka Held-Feindt, Rainer Adelung, Stephan Wulfinghoff, Fabian Schütt

**Affiliations:** 1 Functional Nanomaterials, Institute for Materials Science, Faculty of Engineering, Kiel University, 24143 Kiel, Germany; mhau@tf.uni-kiel.de (M.H.); roma@tf.uni-kiel.de (R.M.); 2 Computational Materials Science, Institute for Materials Science, Faculty of Engineering, Kiel University, 24143 Kiel, Germany; jd@tf.uni-kiel.de; 3 Institute of Metallic Biomaterials, Helmholtz-Zentrum Hereon, Max-Planck-Str. 1, 21502 Geesthacht, Germany; berit.zeller-plumhoff@hzg.de; 4 Department of Neurosurgery, University Medical Center Schleswig-Holstein UKSH, Campus Kiel, 24105 Kiel, Germany; dana.hellmold@uksh.de (D.H.); carolin.kubelt@uksh.de (C.K.); michael.synowitz@uksh.de (M.S.); janka.held-feindt@uksh.de (J.H.-F.)

**Keywords:** drug delivery system, diffusion-controlled, drug reservoir, glioblastoma, computational modelling

## Abstract

Localized therapy approaches have emerged as an alternative drug administration route to overcome the limitations of systemic therapies, such as the crossing of the blood–brain barrier in the case of brain tumor treatment. For this, implantable drug delivery systems (DDS) have been developed and extensively researched. However, to achieve an effective localized treatment, the release kinetics of DDS needs to be controlled in a defined manner, so that the concentration at the tumor site is within the therapeutic window. Thus, a DDS, with patient-specific release kinetics, is crucial for the improvement of therapy. Here, we present a computationally supported reservoir-based DDS (rDDS) development towards patient-specific release kinetics. The rDDS consists of a reservoir surrounded by a polydimethylsiloxane (PDMS) microchannel membrane. By tailoring the rDDS, in terms of membrane porosity, geometry, and drug concentration, the release profiles can be precisely adapted, with respect to the maximum concentration, release rate, and release time. The release is investigated using a model dye for varying parameters, leading to different distinct release profiles, with a maximum release of up to 60 days. Finally, a computational simulation, considering exemplary in vivo conditions (e.g., exchange of cerebrospinal fluid), is used to study the resulting drug release profiles, demonstrating the customizability of the system. The establishment of a computationally supported workflow, for development towards a patient-specific rDDS, in combination with the transfer to suitable drugs, could significantly improve the efficacy of localized therapy approaches.

## 1. Introduction

Drug delivery systems (DDS) for the localized treatment of brain tumors, such as glioblastoma, after surgical resection have emerged as an alternative drug administration route, as they potentially offer an increased efficacy and reduce possible side effects, compared to systemic therapies [1,2,3,4,5,6,7,8]. For this purpose, different designs of DDS have been developed, including nano- and microparticles [9,10,11,12] fibers [13,14], meshes [15,16], wafers [17,18] and (injectable) hydrogels [19,20]. Especially, reservoir-based drug delivery systems (also referred to as depots) facilitate a sustained drug release [21,22]. This means that an initial burst effect is prevented, and the therapeutic level of drug concentration is prolonged [23]. Advancements have also been made towards stimuli-responsive DDS [24,25,26] for GBM therapy, which could deliver the drug on-demand, e.g., in the event of a tumor recurrence. Still, these approaches have not yet led to satisfactory therapy outcomes.

One aspect for the improvement of treatments could be the establishment of patient-specific release kinetics, which are customizable for different settings and drugs. Ideally, the DDS provide a zero-order controlled release, leading to a constant drug concentration, within the therapeutic window, for a sufficiently long period at the tumor site [27]. Another essential aspect in localized therapy, that has only rarely been considered in literature, is that, for applications in vivo, the respective conditions need to be contemplated. The implantation of the DDS, after surgical removal of the tumor, inside the remaining cavity, involves, e.g., the exchange of the cerebrospinal fluid [28,29], resulting in a removal or elimination of the drug. Consequently, reasonable amounts of drug need to be loaded in the DDS and released at effective rates to achieve suitable concentration profiles over long time periods. Here, the drug release rate depends on multiple factors, such as the drug loading, drug-matrix interactions, diffusion coefficients, and degradation rates for biodegrading systems [30]. In this context, the respective fabrication method of the DDS and incorporation of the drug plays a key role, e.g., microspheres are commonly prepared by emulsion methods [31] and fibers by electrospinning [32]. Both techniques require a high degree of control over the formulations and process parameters, in order to achieve high drug loading and precise release rates. There are approaches and strategies to overcome these limitations. Hosseinzadeh et al. [33] developed a novel oil-in-oil emulsion method for the incorporation of temozolomide into microspheres, in order to increase the drug loading efficacy. Ramachandran et al. [34] created a library of electrospun temozolomide-loaded nanofibers, with different release times, and combined these appropriately in one implant to obtain suitable release kinetics. Still, it seems that providing precisely adaptable release kinetics remains a key challenge for advanced therapeutic approaches.

**Figure 1:**
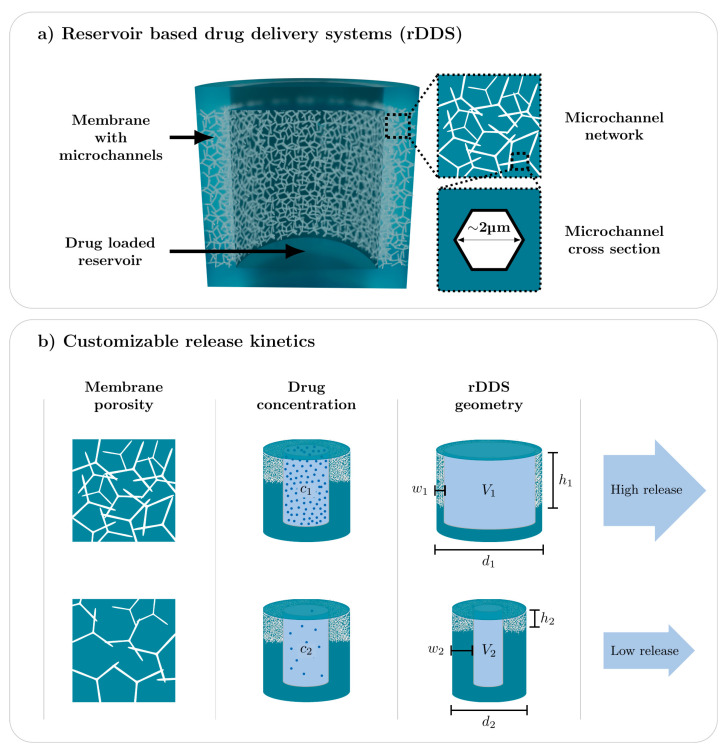
Schematic of the reservoir-based drug delivery system (rDDS) and customizable release kinetics. (**a**) The cylindrical rDDS consists of a reservoir surrounded by a porous membrane, pervaded by a network of hollow microchannels, with a mean diameter of approx. 2 µm. (**b**) The diffusion-controlled release can be tailored by the membrane porosity, drug concentration, and rDDS geometry (membrane width *w* and height *h*, reservoir volume *V*, and sample diameter *d*).

Here, we present the fabrication, characterization, and computational modelling of a reservoir-based, diffusion-controlled drug delivery system (rDDS), with customizable release kinetics. Based on this novel DDS, we demonstrate the workflow for a computationally-supported device development, towards patient-specific drug delivery. The cylindrical rDDS, schematically shown in Figure 1a, consists of a reservoir, surrounded by a porous polymer membrane, containing a network of hollow microchannels (approx. 2 µm mean diameter). The reservoir makes it possible to load considerable amounts of substances, while the membrane is pervaded by a network of interconnected microchannels and determines the diffusion-controlled release. As depicted in Figure 1b, the diffusion-controlled release is tunable by the following parameters: (i) membrane porosity; (ii) drug loading; and (iii) rDDS geometry (membrane width *w* and height *h*, reservoir volume *V*, and sample diameter *d*), thus offering the possibility to precisely and easily adapt the release kinetics. Additionally, the fabrication of the rDDS and subsequent drug loading are separated, facilitating the loading of the rDDS with any substance. Further, we demonstrate the development of a DDS, by support of simulations, making it possible to investigate the influence of defined parameters in detail and promoting a focused fabrication of a suitable system. Exemplarily, the removal of the drug, for example, by the flow of the cerebrospinal fluid, is incorporated into the computational simulation, in order to study the resulting concentration profiles. By this, the simulation can be used to determine required parameters for a specific application scenario.

## 2. Materials and Methods

### 2.1. Fabrication of Reservoir-Based Drug Delivery System

The preparation of the rDDS (Figure S1) is based on a bottom-up fabrication method, presented by Rasch et.al. [35], which utilizes tetrapodal zinc oxide (t-ZnO). The synthesis of t-ZnO is described elsewhere [36,37]. Briefly, zinc powder (Sigma-Aldrich, St. Louis, MO, USA, particle size < 10 μm) and polyvinyl butyral (PVB) (Kuraray) (1:2 mass ratio) were burnt in a furnace, at 900 °C for 30 min, to create tetrapodal-shaped zinc oxide microparticles. The harvested t-ZnO powder was assembled into quadratic sacrificial templates (a = 1 cm, h = 0.5 mm), with a defined density of t-ZnO microparticles (0.25, 0.3, 0.45, 0.55, 0.6 g/cm^3^), by pressing the respective amount of t-ZnO powder into a mold. Subsequently, the t-ZnO templates were sintered at 1150 °C for 5 h, in order to obtain an interconnected network of t-ZnO microparticles. In a next step, the entire free volume of the t-ZnO templates were filled up with polydimethylsiloxane (PDMS) (Sylgard 184). To this end, the elastomer base and curing agent (10:1 mass ratio) were mixed and degassed for 10 min. The t-ZnO templates were placed into a mold with defined height (h = 3 mm or h = 4 mm), and the PDMS mixture was poured over the templates. By keeping the templates under vacuum in a desiccator for at least 30 min, until any bubbles were visible, the PDMS mixture was pushed into the free volume of the t-ZnO template. The molds were filled up completely with PDMS, covered by a lock, and kept for at least 4 h at 80 °C, in order to cure the PDMS. In a following step, a CO_2_ laser-cutter (GS 6090 PU, GS Laser Systems) was applied to manufacture the final sample geometry. First, the scanning mode of the laser-cutter was used to create a reservoir in the t-ZnO/PDMS composite. Finally, cylindrical samples with defined diameters (d = 3 mm or d = 6 mm) were cut out around the reservoirs. The laser-structured samples were kept in hydrochloric acid (HCL) (4 M) for at least one day, in order to etch the t-ZnO, and were then washed thoroughly by exchanging the HCl at least five times with distilled water and three times with ethanol, with at least 1h between the washing steps. By removing the t-ZnO, the density of the sacrificial template translates into a defined porosity of the membrane (5.4, 8.0, 8.9, 9.8, 10.7%). In a final step, the samples were air-dried, and the reservoirs were sealed by a thin film (<200 μm thickness) of PDMS to obtain closed samples. For this, a squeegee iron was used to prepare a thin film of PDMS, which was placed for 8 min in an oven at 80 °C, in order to become sticky. The samples were then placed onto the thin film, and the PDMS was cured for at least 4 h at 80 °C.

### 2.2. Functionalization of Microchannels by Poly(N-Isopropylacrylamide) (pNipam)

The microchannels of three rDDS (d = 6 mm, h = 4 mm, 5.35% membrane porosity) were functionalized by incorporation of poly(N-isopropylacrylamide) (pNipam). For this, a solution with of N-Isopropylacrylamide (2%, 3000 μL), N,N′-Methylenebis(acrylamide) (2%, 500 μL), distilled water (1000 μL), ammonium persulfate (1:10, 100 μL), and N-N-N′-N′-tetramethylethylenediamine (46 μL) was prepared. All chemicals were purchased from Sigma-Aldrich. The samples were immersed in the prepared solution, inside an Eppendorf tube, and degassed for 5 min to infiltrate the microchannels with the solution. The samples were taken out and kept at room temperature for 2 h to allow polymerization.

### 2.3. Characterization of Release Kinetics

Release experiments were performed using aqueous methylene blue (MB) (Sigma-Aldrich) solutions with different concentrations (2.5, 5, 10, 20, 100 mM). The dye was carefully injected into the reservoir using a syringe (0.3 mm) (Figure S2). The microchannels inside the porous membrane were loaded with distilled water by degassing the sample for at least 10 min, inside an Eppendorf tube filled with distilled water, until the porous membrane no longer appeared white, but more translucent. Subsequently, the samples were placed in a cuvette, filled with 3.5 mL of distilled water. The release of the dye was recorded at room temperature by a customized photometer (light source HL-2000-FHSA-LL by Ocean Insight, Ostfildern, Germanyand spectrometer Flame-S-VIS-NIR_ES by Ocean Insight, Ostfildern, Germany), controlled by a customized LabVIEW program (National Instruments, Austin, TX, USA, version 2012), measuring the absorbance of the dye solution inside the cuvette, both automatically and continuously. The solution was diluted, such that the concentration was kept low inside the cuvette, so that the absorbance was not reaching values far above 1. Matlab (The MathWorks Inc., Natick, MA, USA, version R2021a) was used for evaluation of the data. The pNipam-containing rDDS and reference were infiltrated with MB solution (20 mM), using a syringe, and placed in cuvettes containing 3.5 mL distilled water. The MB release was recorded at distinct time points by measuring the absorbance with a UV–VIS spectrometer (Lambda 900, Perkin Elmer, Waltham, MA, USA). After each measurement, the solution was diluted to maintain a low concentration. For regular time intervals of at least 24 h, the cuvettes were placed in water baths and heated to 36 °C.

### 2.4. Scanning Electron Microscopy

Scanning electron microscopy images of rDDS were recorded using a Zeiss Supra 55VP. For this, the samples were sputtered with a thin layer of gold.

### 2.5. Synchroton Radiation-Based Micro Computed Tomography (SRμCT)

#### 2.5.1. Imaging

Four rDDS (d = 3 mm, h = 3 mm), with a nominal membrane porosity of 5.35%, were imaged using synchrotron radiation-based micro computed tomography (SRµCT) at P05 beamline at PETRA III at the Deutsches Elektronen-Synchrotron (DESY, Hamburg, Germany), operated by Helmholtz-Zentrum Hereon. Imaging was conducted using a photon energy of 31 keV, with a field of view of 3.28 × 2.46 mm, and an effective pixel size of 0.64 µm. A total of 4801 projections were obtained, with an exposure time of 550 ms, which were tomographically reconstructed, using bin 2, in a customized reconstruction tool [38], utilizing the ASTRA Toolbox [39,40] in Matlab R2020a (The MathWorks Inc., Natick, MA, USA).

#### 2.5.2. Image Processing and Segmentation

The image was filtered using an iterative non-local means filter [41], with in isotropic search radius of 6 voxel and 4 iterations. The overall rDDS geometry was segmented in Avizo 2020.2 (FEI SAS, ThermoFisher Scientific, Bordeaux, France) using region growing. A closing operation, with a cubic kernel of 3 voxels, in 3D, was performed to close any pores in the PDMS remaining after segmentation. For all evaluation, only a subvolume, of approximately 0.35 mm height of each sample, was selected within the 0.5 mm of theoretical height of the porous network. For the analysis of the PDMS thickness, a Euclidian distance map, based on the segmented reservoir, was obtained and evaluated, only along the outer outline of the overall rDDS. The thickness distribution, as a function of the height along the sample, was then computed and plotted as a boxplot using Matlab R2020a (The MathWorks Inc., Natick, MA, USA). The segmentation of the pore network was performed in Avizo, using thresholding. Using Avizo, the face connectivity of the pore network was calculated using the connected components tool, with a minimum component size of 10 voxels. For further analysis of the porosity, only the largest connected component was considered. The area of the reservoir was computed as the face contact area between reservoir and surrounding PDMS, including pores. Similarly, the interface area of the reservoir and pore network was their face contact area. The overall porosity of the membrane was calculated via the number of voxels of the largest connected component and rDDS, as well as for the whole pore network. Finally, the BoneJ thickness plugin [42] was used to calculate the average pore size via fitting of spheres. The calculation of quantitative parameters, based on voxel number, was performed in Excel 2019 (Microsoft Office, Redmond, WA, USA).

### 2.6. Computational Modelling and Simulation

**Figure 2:**
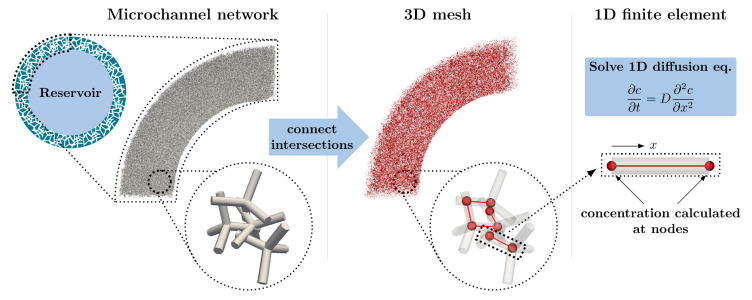
Schematic of the computational model, and simulation of the reservoir-based drug delivery system.

To study the influence of the release-determining parameters and be able to predict the release kinetics, the rDDS was computationally modelled, and release was simulated. In general, diffusion-controlled drug delivery has been modelled and discussed extensively [43,44]. However, the here presented simulation facilitates the investigation of the rDDS with its specific parameters. The approach is schematically shown in Figure 2. The model is based on the observation that the membrane is inverse to the initial t-ZnO template structure, which consists of intersecting tetrapodal particles. Thus, it is possible to obtain an artificial microchannel network by creating an artificial tetrapod network and determining the intersections and connections. By that, a three-dimensional mesh, consisting of one-dimensional elements, is obtained. The tetrapod positions and orientations are chosen as completely random. The tetrapod arm length $l^{\mathrm{ZnO}}$ was chosen as 24 µm, and the arm diameter $d^{\mathrm{ZnO}}$ as 2 µm, corresponding to the typical dimensions of a ZnO tetrapod. The mesh is used in combination with the finite element method (FEM), in order to solve the one-dimensional time dependent diffusion equation along the channels.

Neglecting any internal particle sources (e.g., chemical reactions) and convection, the development of concentration over time is described by Fick’s second law. In the one-dimensional case, it is given by:

(1) $$\frac{\partial c}{\partial t}=D\frac{\partial^{2}c}{\partial x^{2}}\;,\;$$

where c denotes the concentration, t the time, D the diffusion coefficient, and x the position. The weak form of the differential Equation (1) is obtained by multiplication, with a test function (v) and integrating over the volume. In case of a network of interconnected microchannels, the total volume of the network is given by the sum of volumes ($V_{\mathrm{ch}}^{m}$) of each channel with index (m):

(2) $$\overset{N_{\mathrm{ch}}}{\underset{m=1}{\sum }}\overset{l^{m}}{\underset{x=0}{\int }}\left(v\frac{\partial c}{\partial t}+D\frac{\partial^{2}c}{\partial x^{2}}\right)A^{m}\;dx=0.$$

Here, $A^{m}$ is the constant cross section, and $l^{m}$ is the total length of channel m. The total number of channels is given by $N_{\mathrm{ch}}$. Note that the test function (v) is zero at the Dirichlet boundaries and continuous in the network, but otherwise arbitrary. Using partial integration, one obtains:

(3) $$\overset{N_{\mathrm{ch}}}{\underset{m=1}{\sum }}\overset{l^{m}}{\underset{x=0}{\int }}\left(v\frac{\partial c}{\partial t}+\frac{\partial v}{\partial x}D\frac{\partial c}{\partial x}\right)A^{m}\;dx=-\overset{N_{\mathrm{ch}}}{\underset{m=1}{\sum }}{\left[vjA^{m}\right]}_{x=0}^{l^{m}}.$$

Due to particle conservation, the sum of particle currents (J=jA) at each intersection in the network is zero, except at the boundaries. In case of the rDDS, the network has a boundary to the inside reservoir and an outside cavity. It is assumed that the concentration and test function are homogeneous (i.e., spatially constant) at both boundaries. The values are given by $c_{R}$, $c_{C}$, $v_{R}$, and $v_{C}$, respectively. Thus, two particle currents, i.e., $J_{R}$ and $J_{C}$, are defined:

(4) $$\overset{N_{\mathrm{ch}}}{\underset{m=1}{\sum }}\overset{l^{m}}{\underset{x=0}{\int }}\left(v\frac{\partial c}{\partial t}+\frac{\partial v}{\partial x}D\frac{\partial c}{\partial x}\right)A^{m}\;dx=v_{R}J_{R}-v_{C}J_{C}.$$

The particle current from the reservoir into the network ($J_{R}$) is obtained by the rate of change of particles in the reservoir:

(5) $$J_{R}=-\frac{\partial c_{R}}{\partial t}V_{R},$$

where $V_{R}$ is the volume of the reservoir. For the cavity, an additional particle current ($J_{\mathrm{ex}}$), removing particles from the cavity, due to liquid exchange with the surrounding body, is considered. The concentration in the liquid entering the cavity is assumed to be zero. Thus, the particle current from the network into the cavity ($J_{C}$) is given by:

(6) $$J_{C}=\frac{\partial c_{C}}{\partial t}V_{C}+J_{\mathrm{ex}}\;\;\;\;\;\;\;\;\;\;\mathrm{with}\;\;\;\;\;\;\;\;\;\;\;J_{\mathrm{ex}}=c_{C}Q,$$

where $V_{C}$ is the volume of the cavity, and Q is the volume flow of exchanged liquid. Finally, one obtains:

(7) $$\overset{N_{\mathrm{ch}}}{\underset{m=1}{\sum }}\overset{l^{m}}{\underset{x=0}{\int }}\left(v\frac{\partial c}{\partial t}+\frac{\partial v}{\partial x}D\frac{\partial c}{\partial x}\right)A^{m}\;dx=-v_{R}\frac{\partial c_{R}}{\partial t}V_{R}-v_{C}\left(\frac{\partial c_{C}}{\partial t}V_{C}+c_{C}Q\right)$$

Additional information on discretization and FEM implementation, as well as a figure (Figure S4) showing the reproducibility of the meshing algorithm, is given in the SI. The diffusion coefficient (D) of methylene blue was chosen as 6.7 × 10^−6^ cm^2^/s [45]. The meshing was done using Fortran, and the finite element model was implemented in Matlab (The MathWorks Inc., Natick, MA, USA, version R2021a).

## 3. Results and Discussion

### 3.1. Scanning Electron Microscopy and Synchroton Radiation-Based Micro Computed Tomography (SRμCT)

**Figure 3:**
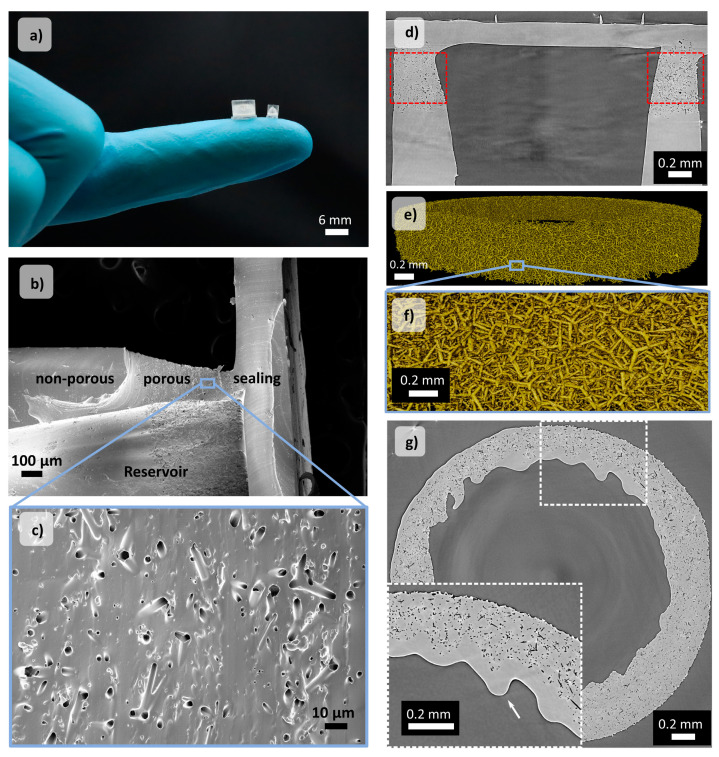
(**a**) Photograph of reservoir-based drug delivery system (rDDS) of different sizes. (**b**) Scanning electron microscopy (SEM) image of the rDDS, showing the non-porous and porous parts of the membrane and the sealing. (**c**) SEM image of the porous membrane. (**d**) Slice from the SRμCT imaging of the rDDS in the yz plane. The red area indicates the sample fraction used for analysis. (**e**) The 3D rendering of the segmented microchannel network and (**f**) zoom into the microchannel network. (**g**) Slice from the SRμCT imaging of the rDDS in the xy plane. The inset shows a zoom into a region where some of the microchannel-reservoir interface has been blocked by PDMS (white arrow).

Figure 3a shows a photograph of two different-sized, reservoir-based drug delivery systems (rDDS) made of polydimethylsiloxane (PDMS). For this study, a standard-sized (d = 3 mm, h = 3 mm, approx. 3.5 µL reservoir volume) and larger rDDS (d = 6 mm, h = 4 mm, approx. 24 μL reservoir volume) were fabricated. The membrane height for both sizes was approx. 400 µm, while the membrane width was kept at either approx. 350 or 800 µm. The preparation of the rDDS (Figure S1) is based on a fabrication method presented by Rasch et al. [35] and combines bottom-up templating and top-down methods. Briefly, a sacrificial template of tetrapodal zinc oxide (t-ZnO) is used to transfer the structure of the interconnected t-ZnO network to the PDMS membrane, leaving behind hollow and interconnected microchannels (see Section 2). By applying the respective density of t-ZnO, a membrane with a defined porosity is obtained. For this study, rDDS, with porosities between 5.4% and 10.7%, were prepared while in principal, it is possible to adjust the membrane porosity between 3.5% up to 48% [35]. Further, it is also feasible to tailor the height of the porous membrane by adjusting the height of the t-ZnO template. The final shape and geometry of the rDDS and the incorporation of a reservoir are created by laser-structuring. In this step, the geometry parameters of the rDDS including the sample diameter, reservoir size and membrane width can be adjusted. The reservoir is finally sealed by a thin PDMS film. Scanning electron microscopy images in Figure 2 show the reservoir, sealing, and zoom into the interconnected network of hollow microchannels inside the membrane. Furthermore, synchrotron radiation-based micro computed tomography (SRµCT) was used to image the rDDS and to investigate the reproducibility of the fabrication method. For this, four samples, with a membrane porosity of 5.3%, were analysed. Figure 3d–g show slices from SRµCT imaging and a 3D rendering of the pore network. As shown, the PDMS membrane thickness varies with the radius and position along the height of the rDDS. All samples show a change in median membrane width of approximately 20% between top and bottom (Figure S3). Figure 3d clearly shows that only part of the rDDS is porous, as designed. The 3D rendering displayed in Figure 3e,f shows the microchannel network resembling the previous t-ZnO structure, with varying sizes of tetrapod arms visible in the picture. The inset in Figure 3g reveals that part of the reservoir was filled by PDMS during the sealing step, so that a fraction of the microchannel-reservoir-interface is covered. This might lead to a reduced reproducibility of the samples. Calculations of decisive sample parameters (Table S1), based on the SRµCT point to further sample differences, which might result in a variance of the diffusion-based release kinetics. Specifically, the microchannel-reservoir surface area fraction deviates significantly by 15% from its mean. This is most likely due to the partial filling of the reservoir with PDMS. Further, the membrane porosity also deviates strongly, by 10%, from its mean. By contrast, the deviations in mean microchannel thickness and microchannel connectivity are low at 5.10% and 3.47% of the mean, respectively.

### 3.2. Chracterization of Release Kinetics

**Figure 4:**
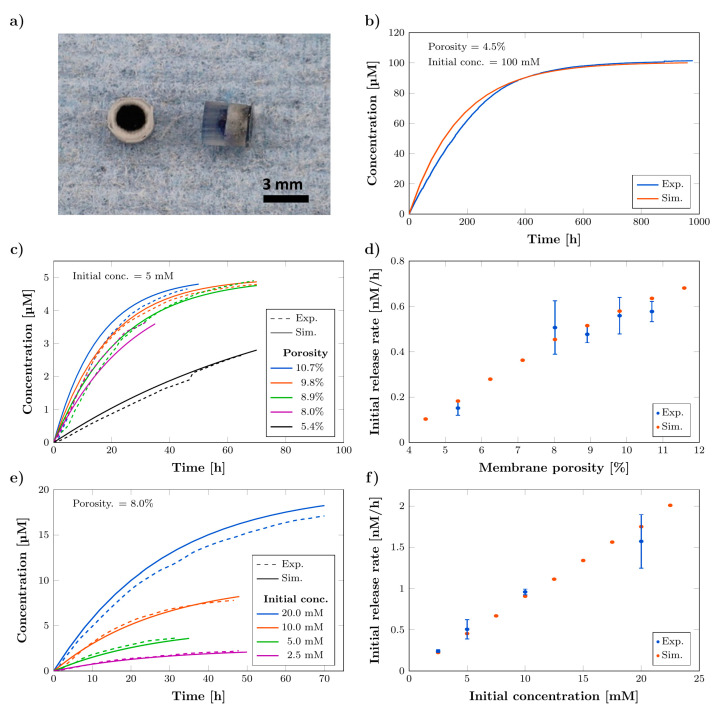
Characterization of release kinetics from standard-sized reservoir-based drug delivery systems (rDDS) with experimental results (Exp.) compared to simulation data (Sim.). (**a**) Photograph of top and side view of rDDS loaded with methylene blue (MB) solution. (**b**) Concentration curve showing a long-term release from rDDS with 4.5% membrane porosity and initial concentration inside the reservoir of 100 mM. (**c**) Concentration curves for varying membrane porosities (5 mM initial concentration) and (**d**) respective initial release rates calculated as mean between 0–20 h. (**e**) Concentration curves for varying initial concentrations (5.4% membrane porosity) and (**f**) respective initial release rates calculated as mean between 0–20 h. All experimental release curves are averages (*n* = 3), and error bars represent the standard deviation.

The release kinetics of the standard-sized reservoir-based drug release system (rDDS) were studied in water, using aqueous solutions of the model dye methylene blue (MB) for different membrane porosities and initial concentrations inside the reservoir. For this, the reservoir of the prepared rDDS was loaded with MB solution via syringe injection (Figure S2), while the microchannels inside the membrane were loaded with distilled water under vacuum. The separate loading of the reservoir and membrane enables both to be filled with different liquids, while their mixing is delayed by the pore structure of the membrane. This results in a reduced burst effect. Figure 4a shows a photograph of a loaded rDDS. Triplicates were measured and the release curves were averaged. All curves are shown in the SI (Figure S5). In Figure 4b, the concentration curve, demonstrating a long-term release from a rDDS with a porosity of 4.5% and initial concentration of 100 mM, is shown, revealing a release for over 400 h and approx. constant release rate for about 200 h. The comparison of the experimental results to the simulation data shows a sufficient agreement. Figure 4c,e show the average concentration curves for different membrane porosities, with an initial concentration of 5 mM inside the reservoir and for varying initial concentrations with a fixed porosity of 8.0%, respectively. Figure 4d,f show the respective initial release rates calculated as mean between 0–20 h from the concentration curves. The results demonstrate the possibility to tailor the release profiles and rates by varying the membrane porosity and initial concentration. Especially, the latter can be easily achieved, in contrast to conventional approaches, as the loading of the rDDS is independent of its fabrication. While we here demonstrate the release from the rDDS for a water-soluble dye, this method could be readily applied to other substances and solvents, depending on the application scenario. In this context, it is crucial to consider the chemical and physical properties of the respective drug, especially with respect to the solubility and diffusion coefficient, as these strongly influence the drug release kinetics. Active substances applied for GBM treatment, such as carmustine [46], temozolomide [47], or paclitaxel [48], are often lipophilic and, thus, need to be combined with suitable solvents. Still, also hydrophilic drugs, such as doxorubicin, have been explored for GBM treatment [49]. However, research has also focused on nanoparticles for delivery of drugs with different properties [50]. Against this background, it would also be interesting to investigate the release of drug-carrying nanoparticles from the rDDS for additional functionalities, such as improved tissue penetration and targeting [51,52], in future work. Besides the properties of the drug, the surrounding environment inside the human body, such as the release medium and temperature, have a great influence on the release kinetics. An increased temperature of 37 °C might affect the rate and total time of drug release. The same holds for the release medium, more precisely the cerebrospinal fluid (CSF), inside the brain. Here, especially the osmolality, salt concentration, and pressure are parameters to influence the drug release. Thus, future release studies should consider simulated in vivo conditions, with respect to the addressed aspects.

Further, the comparison to the simulation results validates the computational modelling. In terms of replicability, the results of the varying membrane porosities indicate a scatter of the initial release rate. This probably results from porosity differences between the samples and is supported by the SRμCT findings, presented above, which revealed a standard deviation of approx. 10% from the mean for the membrane porosity. However, the reproducibility is still sufficient, as the maximum standard deviation of the initial release rate for varying porosities is only approx. 23% of the mean. Here, special emphasis should be placed on the fact, that the three-dimensional micro-structuring of the membrane is achieved by a simple template-approach and requires no complex structuring processes.

### 3.3. Influence of Parameters on Release Kinetics

**Figure 5:**
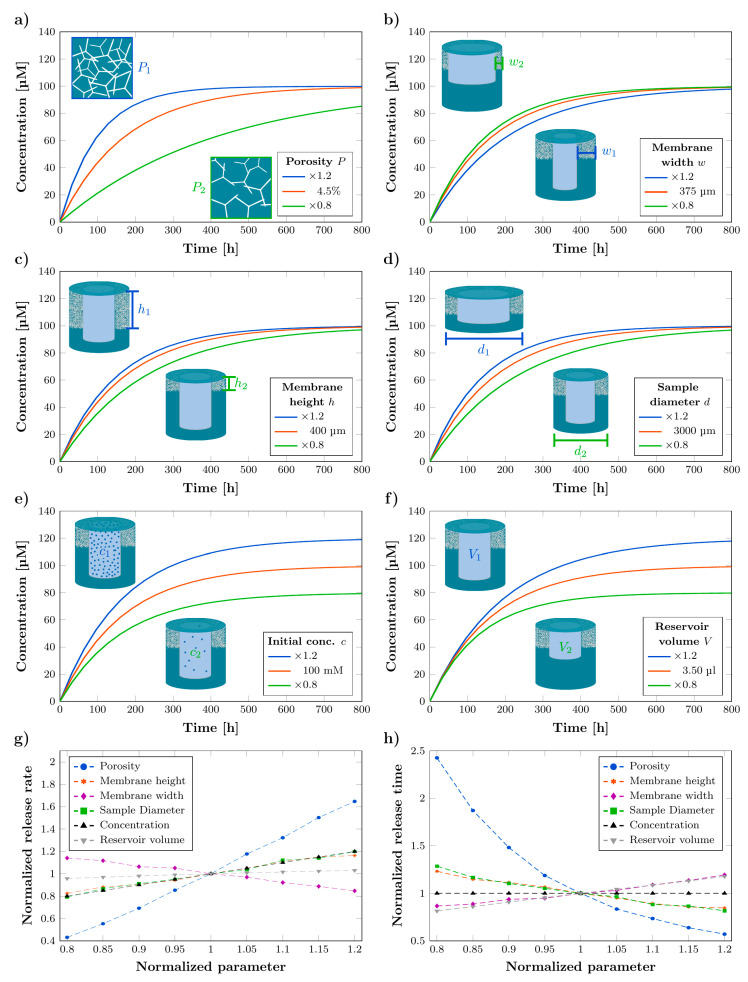
Simulated concentration curves showing the influence on the release kinetics by the (**a**) membrane porosity, (**b**) membrane width, (**c**) membrane height, (**d**) sample diameter, (**e**) initial concentration, and (**f**) reservoir volume. Red curves represent the concentration curve of the standard rDDS (compare Table 1) while blue and green curves show the concentration curves with +20% and −20% deviation of the respective parameter. (**g**) The normalized release rate and (**h**) normalized release time, as a function of the adjustable parameters, normalized to their initial values (compare to Table 1).

The release kinetics can be customized by changing the membrane porosity *P*, membrane width *w* and membrane height *h*, the sample diameter *d*, the initial drug concentration *c* and the reservoir volume $V_{R}$. For characterization, a standard simulation setup summarized in Table 1 was chosen and deviations by ±20% of the six adjustable rDDS parameters were investigated. The cavity volume $V_{C}$ was kept constant at 3.5 mL, as for the experimental tests. Note that only one parameter was changed at a time, while all the others were kept constant. The influences on the concentration curves are depicted in Figure 5a–f. Further, the normalized release rate and normalized release time are given in Figure 5g,h. Release time, in this context, means the amount of time until the concentration in the reservoir has decreased to 50% of the initial concentration. Rate, time, and parameters are normalized to the values of the standard simulation setup. A summary of the results is given in Table 2. Increasing the porosity leads to an increase in release rate by 65% and a decrease in release time by 43% and decreasing the porosity leads to a decrease in release rate by 57% and an increase in release time by 143%. The geometric changes in membrane height, membrane width, and sample diameter by 20% lead to a change in release rate between 14 and 21%, as well as a change in release time between 13 and 29% and, thus, show a significantly smaller impact. It is, therefore, rational to consider the porosity as the major parameter to set the magnitude of the release rate and time while the geometric changes can be used for tuning. Especially interesting, in terms of tailoring the release profiles, are the changes in initial concentration and reservoir volume, as they only influence either the release rate or the release time while keeping the other constant. Note that, in case of the reservoir volume change, the initial release rate at t = 0 s is exactly preserved, but then changes, due to the different concentration developments in the reservoir. Thus, calculating the rate with concentration and time increments, it is not exactly constant. Further, as the diffusion coefficient is included as a parameter in the model, the simulations can be transferred to other substances and specific drugs in different solvents by considering the respective diffusion coefficient.

**Figure 6:**
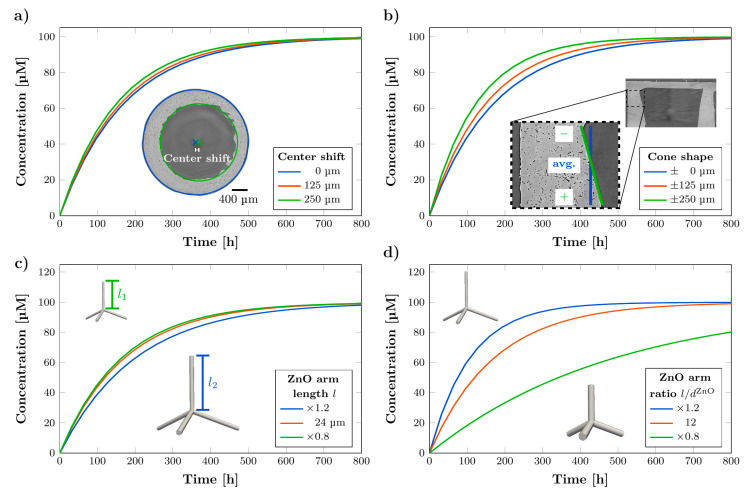
Simulated concentration curves showing the influence on the release kinetics by (**a**) a center shift of the reservoir, (**b**) cone shape of the reservoir, (**c**) size of the tetrapods in terms of the arm length with constant ratio l/d and (**d**) ratio of the arm length to arm diameter of the tetrapods.

In addition to the six controllable parameters there are a few more parameters that potentially influence the release. Figure 6 shows the results of the investigation of these parameters. In the above simulations the reservoir is perfectly centered and has a cylindrical shape. SEM and SRμCT images show that in reality the reservoir may be shifted off center and has often a shape that resembles a truncated cone. In both cases the simulation predicts even for large deviations from the idealized case only a small impact on the release kinetics as shown in Figure 6a,b. A center shift of 250 µm (67% of membrane width) leads to an increase in release rate by 11% and a decrease of release time by 11%. A decrease in membrane width at the top of the membrane and increase at the bottom of the membrane by 250 µm leads to an increase in release rate by 34% and a decrease in release time by 27%. Although for large deviations the impact on release is comparably small, both parameters need to be considered for a high reproducibility of the rDDS release. Furthermore, the membrane properties of the microchannels are influenced by the shape of the sacrificial ZnO tetrapods. For a constant porosity, a higher volume per individual ZnO microparticle means a lower interconnectivity. Indeed, changing the ratio of tetrapod arm length and arm diameter drastically changes the number of connections in the mesh and, thus, the release kinetics, as depicted in Figure 6d. Keeping a constant ratio and changing the size of the particles (while reducing their number to keep the porosity constant), however, has only a comparable small influence as shown in Figure 6c.

### 3.4. Long-Term Release Kinetics

**Figure 7:**
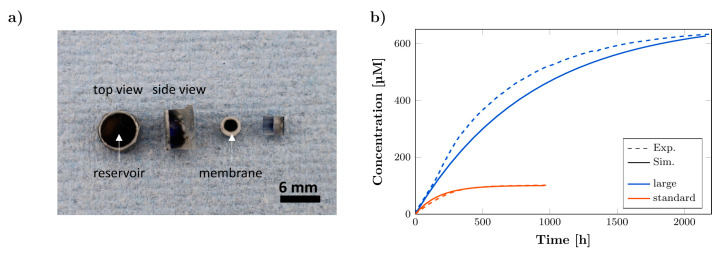
Long-term release kinetics. (**a**) Photograph of top and side view of large and standard-sized rDDS loaded with methylene blue (MB) solution. (**b**) Long-term release from large and standard-sized rDDS with 4.5% membrane porosity and initial concentration inside the reservoir of 100 mM with experimental results (Exp.) compared to simulation data (Sim.). Experimental concentration curves are averages (*n* = 2 for large and *n* = 3 for standard-sized rDDS).

Further, the release of MB was also studied for a large rDDS (d = 6 mm, h = 4 mm, approx. 800 μm membrane width) with 4.5% membrane porosity and an initial concentration of 100 mM. In Figure 7a photograph of large rDDS loaded with MB solution is shown in comparison to standard-sized rDDS. The large rDDS revealed a release for over 1500 h and an approx. constant release for about 500 h at a higher release rate compared to the standard-sized rDDS, shown in Figure 7b. This is due to the larger size and higher amount of MB loading. The comparison to the simulation results reveals a sufficient agreement. The results demonstrate that the fabrication of larger rDDS is possible which could be suitable for larger tumor cavities.

### 3.5. Computational Simulation of Concentration Profiles for Exemplary In Vivo Conditions

**Figure 8:**
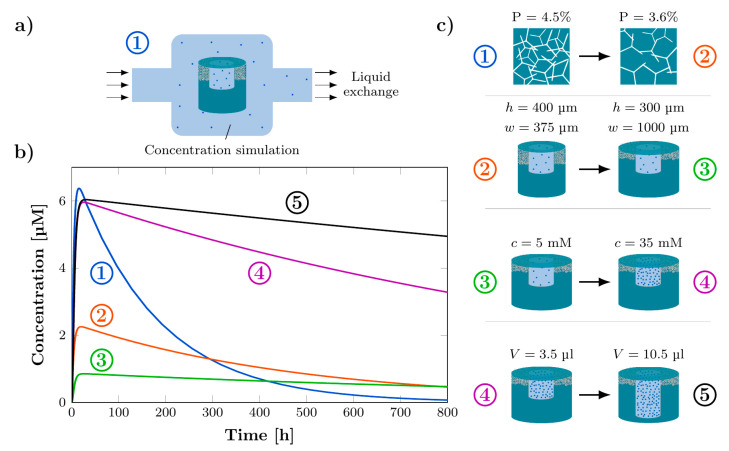
Computational simulation of concentration profiles for exemplary in vivo conditions. (**a**) Schematic of the rDDS inside the tumor cavity releasing the drug, which is transported out of the cavity by the exchange of liquid. (**b**) Simulated concentration profiles for varying parameters. Starting from the standard rDDS (compare Table 1), with 5 mM initial concentration, for the respective subsequent concentration profile, a certain parameter was changed, compared to the previous one. The numbering indicates the order of parameter changes. (**c**) Schematic of the respective parameter changes from one concentration profile to the subsequent one.

While most of the studies only focus on the release into a stationary volume, to simulate the resulting concentration profiles under exemplary in vivo conditions, the removal of the drug, e.g., by exchange of the cerebrospinal fluid, was included in the computational modelling. The alteration of the setup from Table 1 is schematically shown in Figure 8a. The volume of the cavity is decreased to 60 μL and an exchange of 15.8 μL/h liquid in the cavity is assumed. Figure 8b depicts the simulated different concentration profiles for varying parameters while in Figure 8c the respective parameter changes are shown schematically. Starting from the standard rDDS (compare Table 1) with an initial concentration of 5 mM, for the respective subsequent concentration profile a certain parameter was changed compared to the previous one to study the influence on the concentration profile. The parameter changes were selected within the experimentally possible range. The numbering in Figure 8b,c indicates the order of parameter changes. The standard rDDS exhibits a strong increase of the concentration to 6 μM within the first few hours which rapidly decreases again. From number 1 (standard rDDS) to number 2 the membrane porosity was reduced from 4.5% to 3.6% resulting in a reduction of the maximum concentration to 2 μM and a slower decline. By reducing the membrane height from 400 μm to 300 μm and increasing the membrane width from 375 μm to 1000 μm (number 2 to number 3) it is possible to achieve an almost constant concentration of 0.85 μM for 800 h. To reach a higher maximum concentration again the initial concentration was increased from 5 mM to 35 mM (number 3 to number 4) and to obtain a more constant concentration level again, the reservoir volume was tripled (number 4 to number 5). This investigation shows that by varying the different parameters it is possible to achieve specific concentration profiles. This helps to adapt the rDDS for customized release kinetics and to determine the required parameters.

### 3.6. Functionalization of Microchannels by Thermo-Responsive Hydrogel for Stimuli-Responsive System

**Figure 9:**
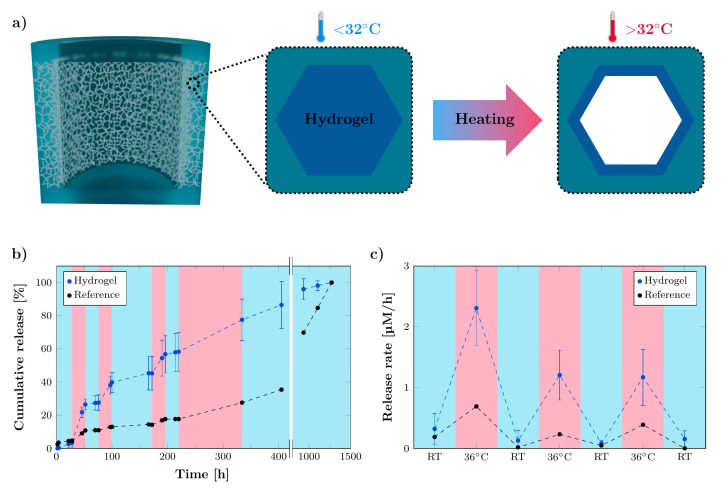
The functionalization of microchannels by thermo-responsive hydrogel for temperature-responsive release. (**a**) Schematic of the rDDS with thermo-responsive hydrogel inside the microchannels. The hydrogel shrinks upon heating above the lower critical solution temperature of 32 °C. (**b**) Cumulative release of methylene blue from hydrogel-containing rDDS, as well as a reference, during multiple heating steps to 36 °C and subsequent cooling to RT. (**c**) Release rates of hydrogel-containing rDDS, as well as the reference, as determined from the cumulative release curve for the respective heating and cooling steps. Data points of hydrogel-containing rDDS are averages (*n* = 3), and error bars represent the standard deviation.

While the here demonstrated rDDS is a passive system, in which the release is purely controlled by diffusion kinetics, the fact that fabrication and loading are separated and can be used to create active rDDS was investigated, e.g., by functionalizing the microchannels inside the membrane. As a proof of concept, the thermo-responsive hydrogel poly(N-isopropylacrylamide) (pNipam) was incorporated into the porous membrane of three large rDDS (d = 6 mm, h = 4 mm, 5.4% membrane porosity, approx. 350 µm membrane width), in order to obtain a stimuli-responsive release system with temperature as a trigger, schematically shown in Figure 9a. The hydrogel pNipam undergoes a phase transition when heated above its lower critical solution temperature (LCST) of around 32 °C from a coil-to-globule state, which results in the release of water, with an accompanied volume decrease [53,54,55]. The phase transition of pNipam is reversible, leading to absorption of the water and reswelling upon cooling below the LCST again. Thus, the incorporation of pNipam into the microchannels could lead to an enhanced or impeded drug release, upon deswelling of the hydrogel at elevated temperatures or reswelling at lower temperatures, respectively. The release of methylene blue (20 mM) was studied from three hydrogel-containing rDDS, as well as a reference containing no hydrogel. For regular time intervals of at least 24 h, the ambient temperature was increased from room temperature (RT) to 36 °C, in order to investigate the effect of temperature on the MB release. Figure 9b shows the cumulative release of MB. The release rates during the consecutive heating and cooling steps are compared between the reference and hydrogel-containing rDDS (Figure 9c). The difference between the rates at RT and during heating is more pronounced for the hydrogel-containing rDDS, compared to the reference. These results demonstrate that the incorporation of pNipam into the microchannels makes it possible to increase and decrease the release rate successively. The functionalization of the microchannels opens up another possibility to further tailor the drug release towards patient-specific release kinetics. However, options, for external or internal triggers, to elevate the temperature need to be researched in detail in future work. As external trigger, e.g., ultrasound, can be used to locally induce a temperature increase [56]. Another possibility is to combine the thermo-responsive hydrogel with nanomaterials, such as graphene or magnetic nanoparticles, that respond to external triggers (e.g., electrical or magnetic fields) [57]. Further, a change in pH, associated with tumor growth, can serve as internal trigger. For this, pH-responsive hydrogels could be incorporated into the microchannels and tested for suitability [58].

## 4. Conclusions

In this work, we presented the development and fabrication of a reservoir-based drug delivery system (rDDS), against the background of localized glioblastoma treatment. The release kinetics of the rDDS were characterized using a model dye. The experimental investigation revealed the precise adjustability of the release profiles via different parameters, such as membrane porosity and initial concentration; additionally, a long-term release up to 60 days was demonstrated. In an outlook, the possibility to functionalize the rDDS from a passive to an active release system was presented. Further, our strategy involves the support of computational modelling and simulation of the system. This enabled the detailed investigation of individual parameter influences on the release profiles. Additionally, the removal of a drug, e.g., by the cerebrospinal fluid exchange, was considered in the simulation, in order to study the resulting concentration profiles. This approach facilitates the customizability of concentration profiles towards patient-specific drug delivery, which could help to increase the treatment efficacy. Future work should focus on the testing of suitable drugs in appropriate models.

## Figures and Tables

**Table 1:** Standard simulation setup.

Parameter	Symbol	Quantity	Schematic
Membrane porosity	*P*	4.5%	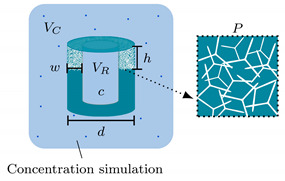
Membrane width	*w*	375 μm	
Membrane height	*h*	400 μm	
rDDS diameter	*d*	3000 μm	
Reservoir volume	*V_R_*	3.5 μL	
Initial concentration	*c*	100 mM	
Cavity volume	*V_c_*	3500 μL	

**Table 2:** Influence of parameter change on release rate and release time.

Parameter	Change	Change in Release Rate	Change in Release Time
Membrane porosity	+20% −20%	+65%	−43%
		−57%	+143%
Membrane width	+20% −20%	−15%	−13%
		+14%	+19%
Membrane height	+20% −20%	+16%	−15%
		−17%	+23%
Sample diameter	+20% −20%	+20%	−18%
		−21%	+29%
Initial concentration	+20% −20%	+20%	0%
		−20%	0%
Reservoir volume	+20% −20%	+3%	−19%
		−4%	+18%

## Data Availability

The data that support the findings of this study and code used for the simulations are freely available on request from the corresponding author.

## Supplementary Materials

The following supporting information can be downloaded at: https://www.mdpi.com/article/10.3390/pharmaceutics14040777/s1. Figure S1: Fabrication of reservoir-based drug delivery system; Figure S2: Loading of reservoir-based drug delivery system; Figure S3: Synchrotron radiation-based μCT analysis of PDMS templates; Figure S4: Mesh reproducibility; Figure S5: Release curves; Table S1: Quantitative PDMS microchannel network analysis.
