# Supplementary material for: Fabrication and Modelling of a Reservoir-Based Drug Delivery System for Customizable Release
Source: Pharmaceutics. 2022 Apr 2;14(4):777. doi: 10.3390/pharmaceutics14040777 (PMC9025308; doi:10.3390/pharmaceutics14040777)
Supplement: Supplementary file 1 [file pharmaceutics-14-00777-s001.zip › pharmaceutics-1650660-supplementary.pdf]

Supplementary Information

Fabrication of reservoir-based drug delivery system

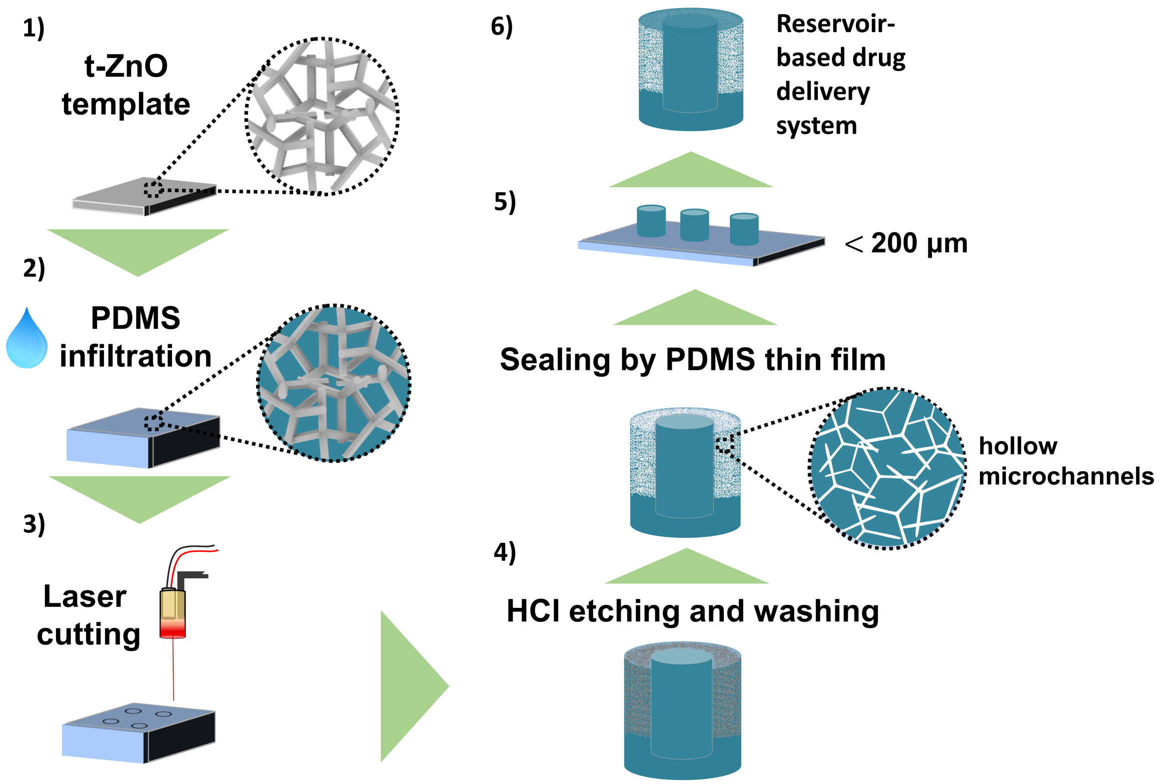

Figure S1: Schematic of fabrication method for reservoir-based drug delivery system including the following steps:1) Preparation of sacrificial template of tetrapodal zinc oxide (t-ZnO), 2) vacuum infiltration of t-ZnO template with polydimethylsiloxane (PDMS), 3) laser cutting of reservoir-containing cylinders, 4) etching of t-ZnO by HCL and subsequent washing of samples to obtain membrane with hollow microchannels, 5) sealing of reservoir by PDMS thin film, 6) final drug delivery system consisting of reservoir surrounded by porous membrane.

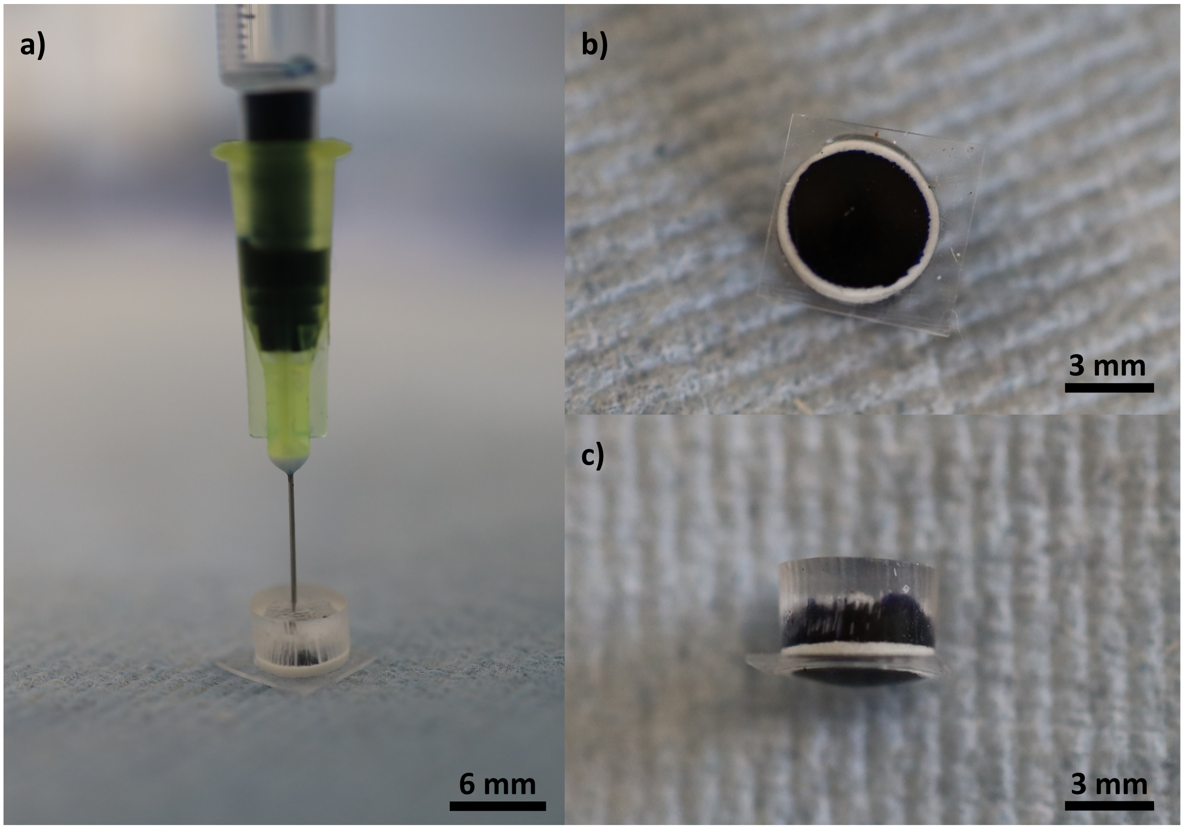

Figure S2: a) Loading of reservoir with methylene blue solution via syringe injection (0.3 mm). b) Top view and c) side view of loaded reservoir-based drug delivery system.

Synchrotron radiation-based µCT Analysis of PDMS templates

Figure S3 shows the variability of the PDMS thickness per sample as a function of its height as a box plot. All samples show a change of median thickness of approx. 20% between top and bottom. This emphasises irregularities from the laser cutting process of the reservoir during manufacturing.

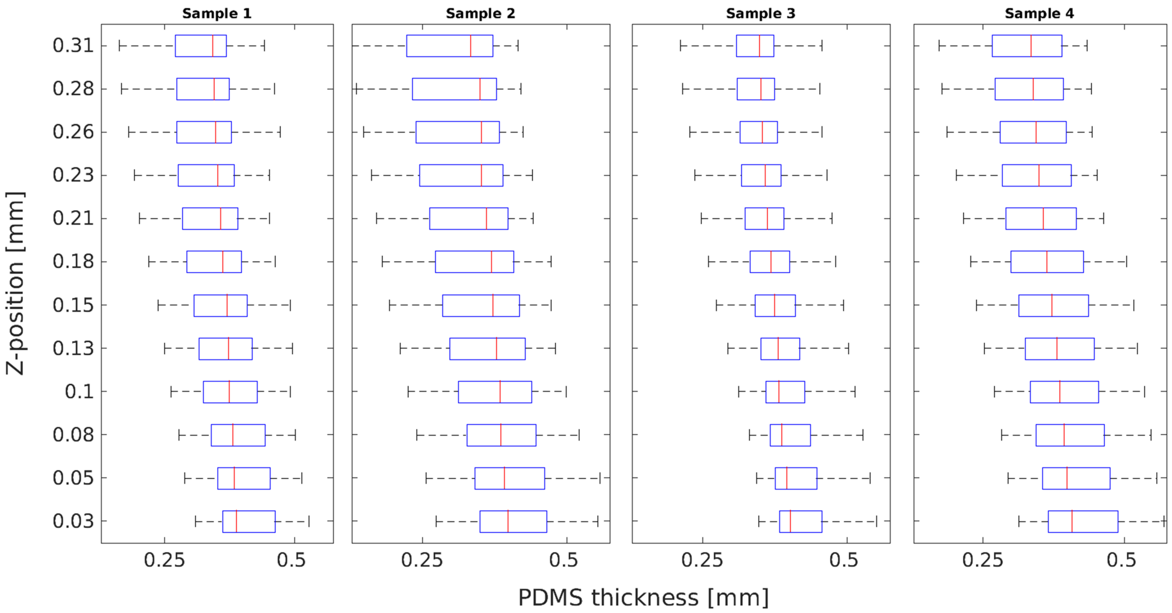

Figure S3: PDMS thickness as a function of the position along the height of the sample for the four rDDS imaged using SRµCT. A decrease in thickness toward the top of the template is visible for all samples. The median thickness from top to bottom deviates by approx. 20%.

Table S1 displays the quantitative analysis of sample porosity, reservoir surface area and microchannel size.

Table S1: Quantitative PDMS microchannel network analysis.

	Mean ± Std
Reservoir surface area [mm^2]	4.30 ± 0.22
Microchannel-reservoir interface area [mm^2]	0.16 ± 0.03
Microchannel-reservoir surface area fraction [%]	3.61 ± 0.54
PDMS porosity (largest connected) [%]	5.94 ± 0.77
PDMS porosity [%]	6.61 ± 0.68
Microchannel connectivity [%]	90.34 ± 3.14
Microchannel thickness [µm]	4.14 ± 0.21

All of the computed parameters are prone to some error due to the segmentation, but as all samples were segmented identically, the segmentation error should be invariant between computed parameters. The quantitative analysis points to a number of reasons for the differences in the release of fluids from the rDDS. While the reservoir surface area standard deviation is less than 5%, the interface surface area and surface area fraction deviate by up to 19% from their mean value which is most likely due to the partial filling of the reservoir with PDMS during the sealing. Sample 4 in particular shows a low surface area fraction of 2.74%, while sample 2 displays the highest fraction at 4.13%.

FEM model

The weak form of the 1D diffusion equation derived in the manuscript is given by:

$$\sum_{m=1}^{N_{\mathrm{ch}}} \int_{x=0}^{l^m} \left(v\frac{\partial c}{\partial t} + \frac{\partial v}{\partial x} D \frac{\partial c}{\partial x} \right) A^m \, \mathrm{d}x = -v_{\mathrm{R}} \frac{\partial c_{\mathrm{R}}}{\partial t} V_{\mathrm{R}} - v_{\mathrm{C}} \left(\frac{\partial c_{\mathrm{C}}}{\partial t} V_{\mathrm{C}} + c_{\mathrm{C}} Q \right), \tag{1}$$

Introducing finite elements with shape functions N that interpolate between the nodes i the concentration in one element e can be given by:

$$c(x,t) = \sum_{i=1}^{N_{\mathrm{en}}} N_i^e(x) c_i^e(t) = \mathbf{N}^{e\top} \mathbf{c}^e; \tag{2}$$

$$\frac{\partial c}{\partial x} = \sum_{i=1}^{N_{\mathrm{en}}} \frac{\partial N_i^e}{\partial x} c_i^e = \mathbf{B}^{e\top} \mathbf{c}^e. \tag{3}$$

Choosing one dimensional linear elements with two nodes ($N_{\mathrm{en}} = 2$), the vectors $\mathbf{N}^e$, $\mathbf{B}^e$ and $\mathbf{c}^e$ are given by:

$$\mathbf{N}^e = \begin{pmatrix} N_1^e(x) \\ N_2^e(x) \end{pmatrix}; \qquad \mathbf{B}^e = \begin{pmatrix} \partial N_1^e/\partial x \\ \partial N_2^e/\partial x \end{pmatrix}; \qquad \mathbf{c}^e = \begin{pmatrix} c_1^e \\ c_2^e \end{pmatrix} \tag{4}$$

The function $v(x)$, its derivative as well as the vectors $\mathbf{v}^e$ and $\mathbf{x}^e$ are given in analogy to eqs. (2) to (4). Further, introducing a reference element in a separate coordinate ξ is, which is mapped to x by the Jacobian $J = \partial x/\partial \xi$, one obtains:

$$c(\xi,t) = \mathbf{N}^\top \mathbf{c}^e; \qquad \frac{\partial c}{\partial \xi} = \mathbf{B}^\top \mathbf{c}^e; \qquad \mathbf{B}^e = J^{e-1} \mathbf{B} \tag{5}$$

with

$$\mathbf{N} = \begin{pmatrix} N_1(\xi) \\ N_2(\xi) \end{pmatrix}; \qquad \mathbf{B} = \begin{pmatrix} \partial N_1/\partial \xi \\ \partial N_2/\partial \xi \end{pmatrix}. \tag{6}$$

The shape functions of the reference element with $-1 \leq \xi \leq 1$ are given by:

$$N_1(\xi) = \frac{1}{2}(1-\xi); \qquad N_2(\xi) = \frac{1}{2}(1+\xi) \tag{7}$$

and the Jacobian J^e is obtained by:

$$x(\xi) = \mathbf{N}^\top \mathbf{x}^e; \qquad \frac{\partial x}{\partial \xi} = \mathbf{B}^\top \mathbf{x}^e = J^e. \tag{8}$$

Now it is possible to define the element damping matrix $\mathbf{C}^e$ and element stiffness matrix $\mathbf{K}^e$ as follows:

$$\mathbf{C}^e = \int_{\xi=-1}^{1} A\mathbf{N}\mathbf{N}^\top J^e \mathrm{d}\xi \qquad \approx \sum_{p=1}^{N_{\mathrm{ip}}} A\mathbf{N}(\xi_p)\mathbf{N}^\top(\xi_p) J^e(\xi_p) w_p; \tag{9}$$

$$\mathbf{K}^e = \int_{\xi=-1}^{1} DA\mathbf{B}\mathbf{B}^\top J^{e-1} \mathrm{d}\xi \quad \approx \sum_{p=1}^{N_{\mathrm{ip}}} DA\mathbf{B}(\xi_p)\mathbf{B}^\top(\xi_p) J^{e-1}(\xi_p) w_p. \tag{10}$$

Here, the integral is approximated by a summation over integration points with index p and weight w_p. The position in the reference element is given by ξ_p and the number of integration points is denoted by N_{ip}. Now, eq. (1) can be reformulated as follows:

$$\sum_{e=1}^{N_{\mathrm{el}}} \mathbf{v}^{e\top} \mathbf{C}^e \frac{\partial \mathbf{c}^e}{\partial t} + \sum_{e=1}^{N_{\mathrm{el}}} \mathbf{v}^{e\top} \mathbf{K}^e \mathbf{c}^e = -v_{\mathrm{R}} \frac{\partial c_{\mathrm{R}}}{\partial t} V_{\mathrm{R}} - v_{\mathrm{C}} \left(\frac{\partial c_{\mathrm{C}}}{\partial t} V_{\mathrm{C}} + c_{\mathrm{C}} Q \right) \tag{11}$$

Introducing a connectivity matrix $\mathbf{L}^e$ that maps the local nodes of the element to the global nodes and two line vectors $\mathbf{L}^{\mathrm{R}}$ and $\mathbf{L}^{\mathrm{C}}$ it is possible to write:

$$\mathbf{v}^e = \mathbf{L}^e\mathbf{v}; \qquad v_{\mathrm{R}} = \mathbf{L}^{\mathrm{R}}\mathbf{v}; \qquad v_{\mathrm{C}} = \mathbf{L}^{\mathrm{C}}\mathbf{v}; \tag{12}$$

$$\mathbf{c}^e = \mathbf{L}^e\mathbf{c}; \qquad c_{\mathrm{R}} = \mathbf{L}^{\mathrm{R}}\mathbf{c}; \qquad c_{\mathrm{C}} = \mathbf{L}^{\mathrm{C}}\mathbf{c}; \tag{13}$$

where $\mathbf{c}$ and $\mathbf{v}$ contain the concentration and test function values at the global nodes and

$$\mathbf{C} = \left(\sum_{e=1}^{N_{\mathrm{el}}} \mathbf{L}^{e\top}\mathbf{C}^e\mathbf{L}^e\right) + \mathbf{L}^{\mathrm{R}\top}\mathbf{L}^{\mathrm{R}}V_{\mathrm{R}} + \mathbf{L}^{\mathrm{C}\top}\mathbf{L}^{\mathrm{C}}V_{\mathrm{C}}; \tag{14}$$

$$\mathbf{K} = \left(\sum_{e=1}^{N_{\mathrm{el}}} \mathbf{L}^{e\top}\mathbf{K}^e\mathbf{L}^e\right) + \mathbf{L}^{\mathrm{C}\top}\mathbf{L}^{\mathrm{C}}Q. \tag{15}$$

Note that all element nodes on the same boundary are mapped to the same global node. With that one can reformulate eq. (11) as follows:

$$\mathbf{v}^\top\left(\mathbf{C}\frac{\partial\mathbf{c}}{\partial t} + \mathbf{K}\mathbf{c}\right) = 0 \tag{16}$$

Finally the discretization in time is done as follows:

$$\frac{\partial\mathbf{c}^e}{\partial t} \approx \frac{\Delta\mathbf{c}^e}{\Delta t} = \frac{\mathbf{c}^e_{\mathrm{n}+1} - \mathbf{c}^e_{\mathrm{n}}}{\Delta t} \tag{17}$$

where the index n indicates known quantities from the previous time step and the index $n+1$ indicates quantities of the current time step. The latter is omitted from here on for simplicity. Thus, one has:

$$\mathbf{v}^\top\left(\frac{1}{\Delta t}\mathbf{C} + \mathbf{K}\right)\mathbf{c} = \mathbf{v}^\top\left(\frac{1}{\Delta t}\mathbf{C}\mathbf{c}_{\mathrm{n}}\right). \tag{18}$$

For the model of the rDDS no Dirichlet-BC are applied, which finally leads to:

$$\left(\frac{1}{\Delta t}\mathbf{C} + \mathbf{K}\right)\mathbf{c} = \frac{1}{\Delta t}\mathbf{C}\mathbf{c}_{\mathrm{n}}. \tag{19}$$

Mesh reproducibility

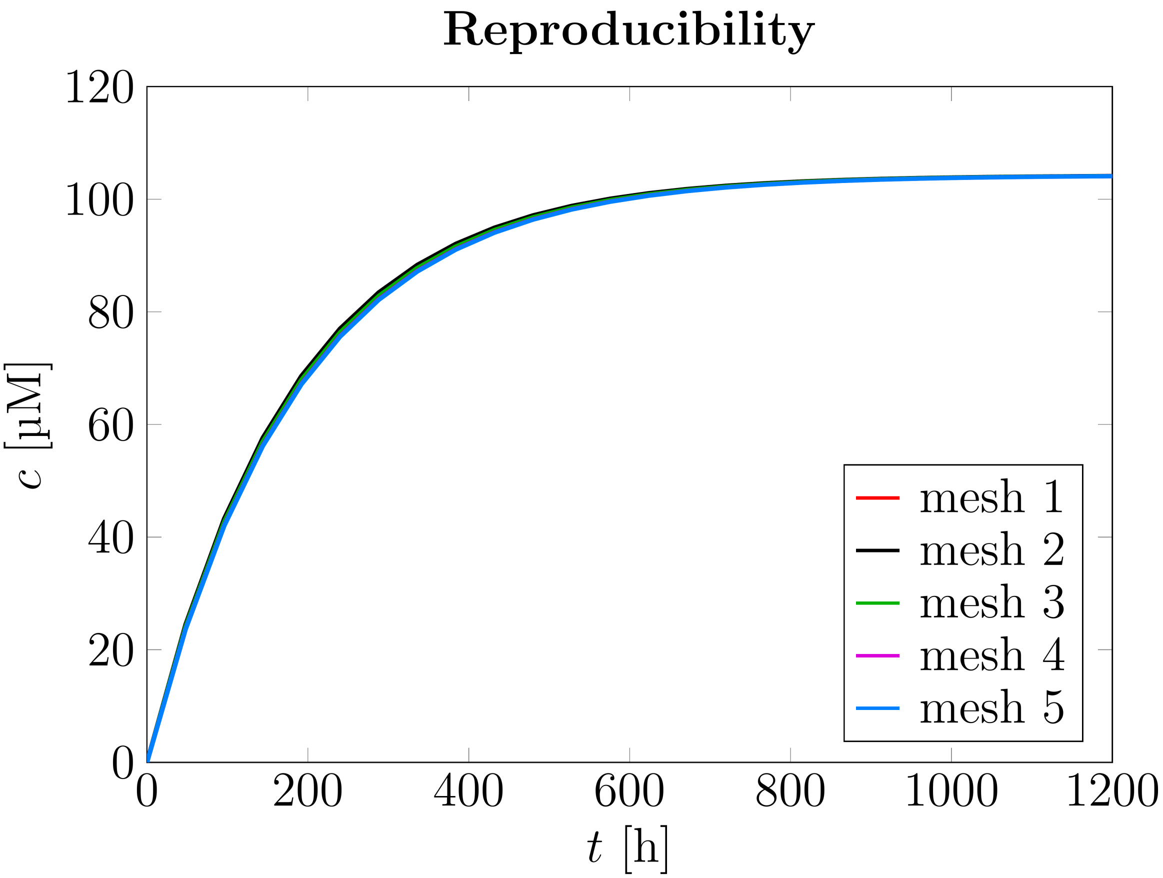

Figure S4: Simulation of release curves using 5 different meshes demonstrating the reproducibility of the meshing algorithm.

Release curves

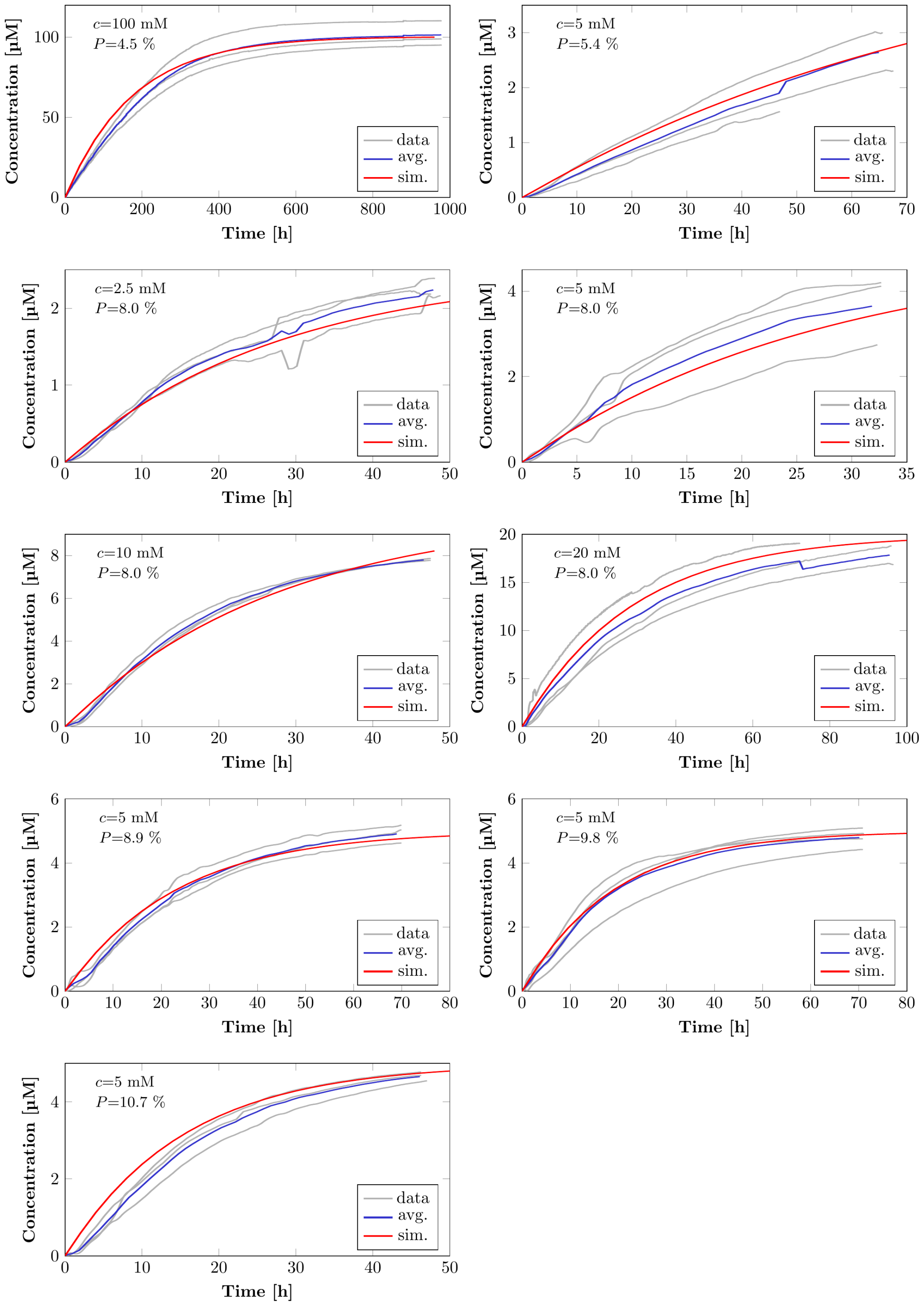

Figure S5: Release curves of methylene blue from reservoir-based drug delivery system with varying porosities and initial concentrations. Blue curves are the average of all measured samples (grey curves) and red curves are the respective simulated release curves.
